# Gold Nanocage-Incorporated Poly(ε-Caprolactone) (PCL) Fibers for Chemophotothermal Synergistic Cancer Therapy

**DOI:** 10.3390/pharmaceutics11020060

**Published:** 2019-02-01

**Authors:** Ju Hyang Park, Hojun Seo, Da In Kim, Ji Hyun Choi, Jin Ho Son, Jongbok Kim, Geon Dae Moon, Dong Choon Hyun

**Affiliations:** 1 Department of Polymer Science and Engineering, Kyungpook National University, Daegu 41566, Korea; pjh99279@naver.com (J.H.P.); dada7230@naver.com (D.I.K.); jode1532@naver.com (J.H.C.); mlbask7@naver.com (J.H.S.); 2 Dongnam Regional Division, Korea Institute of Industrial Technology, Busan 46938, Korea; dark911205@gmail.com; 3 Department of Materials Science and Engineering, Kumoh National Institute of Technology, Gumi, Gyeongbuk 39177, Korea; jb1956k@gmail.com

**Keywords:** PCL, electrospinning, combination therapy, photothermal therapy, NIR-triggered drug release

## Abstract

This paper introduces a new fibrous system for synergistic cancer therapy, which consists of gold nanocage (AuNC)-loaded poly(ε-caprolactone) (PCL) fibers with encapsulation of a chemotherapeutic anticancer drug in their core and loading of a phase-changeable fatty acid in their sheath. Under on–off switching of near-infrared (NIR) light irradiation, the excellent photothermal ability and photostability of AuNCs allows repeated, significant heating of the fibers to a temperature available to hyperthermia. Simultaneously, the NIR light-induced heat generation enables the melting out of the loaded fatty acid, leading to a rapid release of the drug molecules from the fibers. The combination of this NIR light-triggered drug release with the repeated hyperthermia treatment exhibits excellent anticancer efficacy.

## 1. Introduction

Over the past several decades, considerable effort has been dedicated to the fabrication of polymeric fibers. These have a large surface area and high aspect ratio, and are applicable to a broad spectrum of applications, including air filtration, catalysis, energy harvesting and conversion, electronics, sensors, and in regenerative medicine [1,2,3,4,5,6,7]. Among many strategies that have been developed for the production of polymeric fibers, electrospinning has been extensively explored due to its merits such as simplicity, high efficiency, low cost, and high reproducibility [3]. More importantly, it allows a variety of functional molecules to be easily loaded into polymeric fibers [8], which has expanded the applicability of the fibers to the biomedical treatment of diseases through the delivery and even the controlled release of drugs [9].

Recently, the use of electrospun polymeric fibers in biomedical application has been directed towards the therapy of cancer, an intractable health issue worldwide. Such electrospun fibres possess unique features including high drug loading efficiency, good stability as a bulk material, and ease in temporal release of drugs [10,11,12,13,14]. In particular, their implantability at tumor sites or in the cavities remaining after surgical removal of solid tumors enables a localized release of chemotherapeutic drugs, which can maintain a low concentration of the systematic drugs while allowing their sufficient dosage locally [12]. However, the use of chemotherapeutic drugs can bring inevitably several problems such as drug resistance and toxicity in side effects [13,15,16]. Furthermore, it is difficult to sufficiently eliminate tumors using drugs only, even though they are useful for treating many types of cancers. 

In addressing these issues, combination therapy, which uses the synergistic effects of two or more treatment modalities, has been developed for higher therapeutic efficacy. Photothermal therapy (PTT), in which an incident light is converted into heat for destroying cancer cells, has been used together with chemotherapy because it can induce a selective death of cancer cells [17,18]. As the combination strategy, until recently, electrospun polymeric fibers have been incorporated with both chemotherapeutic drugs and photothermal agents. As an exemplary case, smart electrospun fibers, which can potentially realize a synergistic effect of chemotherapy and PTT, were produced by Burdick and coworkers [19]. They encapsulated gold nanorods (AuNRs), as a photothermal agent, and drug molecules into poly(*N*-isopropylacrylamide-co-polyethylene glycol acrylate) (PNPA) fibers with thermosensitivity. Near-infrared (NIR) light-triggered release of the loaded drug occurred by an interplay of the photothermal effect of AuNRs with the temperature responsiveness of PNPA. Mao and coworkers fabricated Yb^3+^/Er^3+^ co-doped CaTiO_3_ nanofibers [12]. Increase in local temperature at the surface of the fibers by NIR light irradiation enhanced the vibration of poly(acrylic acid) (PAA) chains functionalized on the surface of the fibers, weakening an electrostatic bonding between the fibers and preloaded doxorubicin anticancer drug molecules. As a result, NIR light-triggered release of the drug was achieved. Recently, Kim and coworkers demonstrated the use of poly(ε-caprolactone) (PCL) fibers functionalized by NIR light-responsive polypyrrole (PPy) for synergistic cancer therapy [13]. Under an on–off operation of NIR light, the fibers allowed a precisely controlled release of paclitaxel anticancer drug together with a repeated application of NIR light-triggered hyperthermia, which exhibited excellent anticancer efficacy. Nevertheless, for their practical application in cancer therapy, the systems need to stop the undesirable drug release in non-triggered state and/or the loss of activity of chemotherapeutic drugs arising from the use of multi-stage physicochemical processes [20]. Alternatively, a system, consisting of PCL hollow fibers with entrapment of indocyanine green (ICG) in their sheath, was developed for combination therapy [21]. However, the use of ICG as a photothermal agent would make the repeated hyperthermia of the system difficult due to its loss of photothermal ability by photobleaching or chemical/thermal denaturation. 

In this study, we introduce a PCL fibrous system incorporated with gold nanocages (AuNCs) that can function as a NIR light-absorbing photothermal agent with excellent stability. The incorporation of AuNCs enables a significant heating of the fibers upon exposure to NIR light. This NIR light-triggered heat generation can be repeatedly achieved through an on–off switching of NIR light irradiation, leading to a significant death of cancer cells by repeated hyperthermia treatment. Additional loading of the anticancer drug doxorubicin and a phase-changeable fatty acid to the fibres will enhance the anticancer activity of the system more through NIR light-triggered release of the drug in combination with hyperthermia. 

## 2. Materials and Methods

### 2.1. Materials

The following chemicals were purchased from Sigma-Aldrich (St. Louis, MO, USA): PCL (Mn ≈ 45,000 Da), WST-1 solution, doxorubicin hydrochloride (DOX), Span 80, silver trifluoroacetate (CF_3_COOAg), sodium hydrosulfide hydrate (NaHS), poly(vinylpyrrolidone) (PVP, Mw ≈ 55,000 Da), lauric acid (LA), stearic acid (SA), *O*-[2-(3-mercaptopropionylamino)ethyl]-*O*’-methylpolyethylene glycol (PEG-thiol, Mw ≈ 5000 Da), and gold (III) chloride trihydrate (HAuCl_4_·3H_2_O). Ethylene glycol (EG) and sodium chloride (NaCl) were obtained from J.T. Baker (Phillipsburg, NJ, USA) and Duksan Chemical (Daegu, Korea), respectively. Human breast cancer SK-BR-3 cells and fibroblast (FB) cells were procured from Korea Cell Line Bank (KCLB, Seoul, Korea). 

### 2.2. Preparation of Eutectic Mixture of Fatty Acids

We prepared a eutectic mixture of fatty acids with a melting point of 39 °C as previously reported [22]. Briefly, 0.8 g of LA and 0.2 g of SA were first put together in a glass vial (20 mL), followed by heating at 90 °C in nitrogen (N_2_) atmosphere with magnetic stirring at 500 rpm. After 1 h, the fatty acid mixture was cooled down to 25 °C in ambient air. 

### 2.3. Synthesis and Phase Transfer of AuNCs

First, EG (50 mL) was added to a 250 mL round bottom flask and then heated at 150 °C under magnetic stirring using a heating mantle, followed by the addition of a NaHS solution in EG (0.6 mL, 3 mM). After 4 min, 5 mL of a HCl solution in EG (3 mM) was added, followed by 12.5 mL of a PVP solution in EG (20 mg/mL). Then, 4 mL of CF_3_COOAg solution in EG (282 mM) was additionally injected to synthesize silver nanocubes (AgNCs). After 30 min, the reaction was quenched in an ice-water bath, followed by centrifugation and washing with acetone and deionized (DI) water (18.2 MΩ). The resultant AgNCs were converted into AuNCs using a previously reported procedure with several minor modifications [23]. In a typical synthesis process, an aqueous solution of HAuCl_4_ (0.75 mM) was prepared. A specific amount of the HAuCl_4_ solution was then added dropwise into 5 mL of an aqueous solution of AgNCs (0.01 mg/mL) preheated to 90 °C. After 10–15 min, the sample was cooled down to 25 °C and a sufficient amount of NaCl was added to remove the AgCl precipitated in the synthesis. The sample was then centrifuged three times at 9000 rpm for 20 min. The collected AuNCs were re-dispersed in DI water and stored in the dark until further use. 

To achieve phase transfer of the as-synthesized AuNCs from water phase to organic, their surface was modified. In order to accomplish this, 1 mg of the as-synthesized AuNCs was dispersed in 5 mL of DI water, followed by adding 1 mL of an aqueous solution of PEG-thiol (1 wt%) and 3 mL of chloroform. The solution mixture was mechanically agitated using a vortex mixer for 2 min, and thus the AuNCs were transferred from the water phase to the chloroform. The chloroform phase was then extracted, followed by centrifugation, washing with fresh chloroform, and drying in ambient air. The resultant sample was stored at 4 °C until further use. 

### 2.4. Fabrication of Electrospun Fibers 

To fabricate electrospun fibers, a water-in-oil (W/O) emulsion was first prepared. To accomplish this, a Span 80 solution in chloroform was made by dissolving 2 g of the surfactant in 38 g of the solvent. The solution was mixed with 200 μL of an aqueous solution containing DOX (3 mg) to generate a W/O emulsion using a high-speed mixer at a rotation rate of 15,000 rpm. The resultant W/O emulsion (3.5 g) was mixed with the eutectic mixture of fatty acids, the phase-transferred AuNCs, and PCL (1.5 g). The weight fraction of the eutectic mixture to PCL (*f_fatty_*) was varied. Polymeric fibers loaded with AuNCs and DOX were produced by electrospinning the prepared W/O emulsion. For the electrospinning, the W/O emulsion was filled into a syringe (5 mL) with attachment to a stainless steel needle (22 gauge) and a voltage in the range of 6 and 8 kV was applied to the needle. The feed rate of the emulsion was fixed at 0.03 mL/h and an electrically grounded aluminum foil, placed 20 cm away from the needle, was used as a collector. Loading of DOX into the fibers was confirmed using a confocal laser scanning microscope (CLSM) (LSM700, Carl Zeiss, Oberkochen, Germany) and the amount of the loaded drug was quantified through UV–VIS spectral measurement (T60, PG Instrument, Leicestershire, United Kingdom), as previously reported [21].

### 2.5. NIR Light-Induced Heat Generation

The photothermal ability of the as-synthesized AuNCs and the phase-transferred AuNCs was characterized using a digital thermometer (Scilab Co., Daejeon, Korea). In a typical procedure, 0.5 mg of the particles was dispersed in 5 mL of dispersion medium (DI water for the former and chloroform for the latter). Each sample was exposed to 1 W/cm^2^ NIR light with a wavelength of 808 nm (LASERLAB Co., Anyang, Korea) for 50 min. For checking the photothermal behavior of the AuNC-loaded fibers, a mesh made of 20 mg of the fibers with a dimension of 1 cm × 1 cm was first prepared, followed by exposing to NIR light irradiation (1 W/cm^2^). The thermal infrared images and the temperature change were obtained with the use of a thermal infrared camera (G100EX, Avio, Tokyo, Japan). 

### 2.6. Release of DOX from Fibers in Response to NIR Light Irradiation

To obtain the release profiles of DOX, we prepared 100 mg of AuNC-loaded PCL fibers encapsulating DOX, followed by immersing in 5 mL of phosphate-buffered saline (PBS) solution at pH 7.4. After incubation in a thermostat preheated to 37 °C for 4 h under mild agitation, NIR light irradiation (1 W/cm^2^, 808 nm) was applied to the sample for 5 min to increase the temperature to 45 °C. After the treatment, the sample was re-incubated at 37 °C. At different time intervals, 1 mL of the solution was taken out and the concentration of DOX in it was characterized using UV–VIS spectrophotometry, as previously reported [21]. After characterization, the solution was poured back for further testing. For each time interval, three samples were tested. 

### 2.7. Cell Viability Test

The cytotoxicity of NIR light irradiation (1 W/cm^2^, 808 nm) was examined using a previously reported procedure [20]. Additionally, we investigated the cytotoxicity of phase-transferred AuNCs, which was achieved by introducing a solution of the particles (10 μL) in dimethyl sulfoxide with a certain concentration to the cells cultured through the aforementioned procedure. The sample without the treatment with the nanoparticles and NIR irradiation was used as a control. The cell viability was determined using WST-1 assay. 

The biocompatibility of plain PCL fibers, AuNC-incorporated PCL fibers, AuNC-incorporated PCL fibers loaded with DOX, and AuNC-incorporated PCL fibers with loading of the fatty acid mixture and DOX was also tested using FB cells. Meshes with a dimension of 1 cm × 1 cm were first prepared from each type of fibers (20 mg) and then placed in each well of a 24-well cell culture plate. After their sterilization for 30 min under UV light irradiation, FB cells were cultured on the meshes as reported previously [20]. The anticancer activity of those fibers and PCL fibers loaded with the fatty acid mixture and DOX against SK-BR-3 cells was evaluated using a live/dead cell assay as well as a WST-1 assay. SK-BR-3 cells cultured on the meshes made of each type of fibers through the aforementioned procedure were subsequently treated with 1 W/cm^2^ NIR light (5 min of exposure per treatment), followed by incubation at 37 °C for an additional 19–24 h according to the number of the exposure to conduct the WST-1 assay and the live/dead assay using a commercially available kit (Biotium, Fremont, CA, USA). 

### 2.8. Characterization 

Transmission electron microscope (TEM) analysis was performed on the fibers incorporated with AuNCs and the as-synthesized AuNCs using a HT7700 (Hitachi) operated at 75 kV. The morphology of the produced fibers was also investigated using a scanning electron microscope (SEM) (SU8220, Hitachi, Tokyo, Japan) with an accelerating voltage of 3 kV. Differential scanning calorimetry (DSC) measurement was conducted using DSC Q100 (TA Instrument, New Castle, DE, USA). Typically, samples with a weight of approximate 3 mg were used for scanning in the range of −70 and 250 °C at a scanning rate of 2 °C/min. X-ray diffraction (XRD) patterns were recorded in the range of 2θ = 10° to 90° using a Rigaku D/MAX II X-ray diffractometer with Cu K_α_ radiation. 

### 2.9. Statistical Analysis

Student’s *t*-test was used to examine the differences between two groups, and a one-way analysis of variance (ANOVA) with a Tukey’s post hoc test was performed to examine the differences among four groups. *p* < 0.01 was considered to indicate a statistically significant difference. 

## 3. Results and Discussion

NIR light, which has the minimum absorption by human blood and body tissues, is known to penetrate into soft tissues up to several inches in depth and to be converted into heat with a photothermal agent [24], which makes it advantageous for PTT. To achieve NIR light-induced cancer therapy, a NIR light-sensitive system was made of biocompatible/ biodegradable PCL fibers incorporated with AuNCs. The unique plasmonic property of AuNCs due to an interaction of light with their conduction band electrons allows photon energy to be efficiently converted into thermal energy [25]. When the generated heat increases the temperature of the system to a window available for hyperthermia (ca. 40–45 °C) [26], inactivation and eventual death of cancer cells will occur. Since AuNCs are non-susceptible to photobleaching and chemical/thermal denaturation [27], the hyperthermia can be repeatedly achieved. 

**Figure 1:**
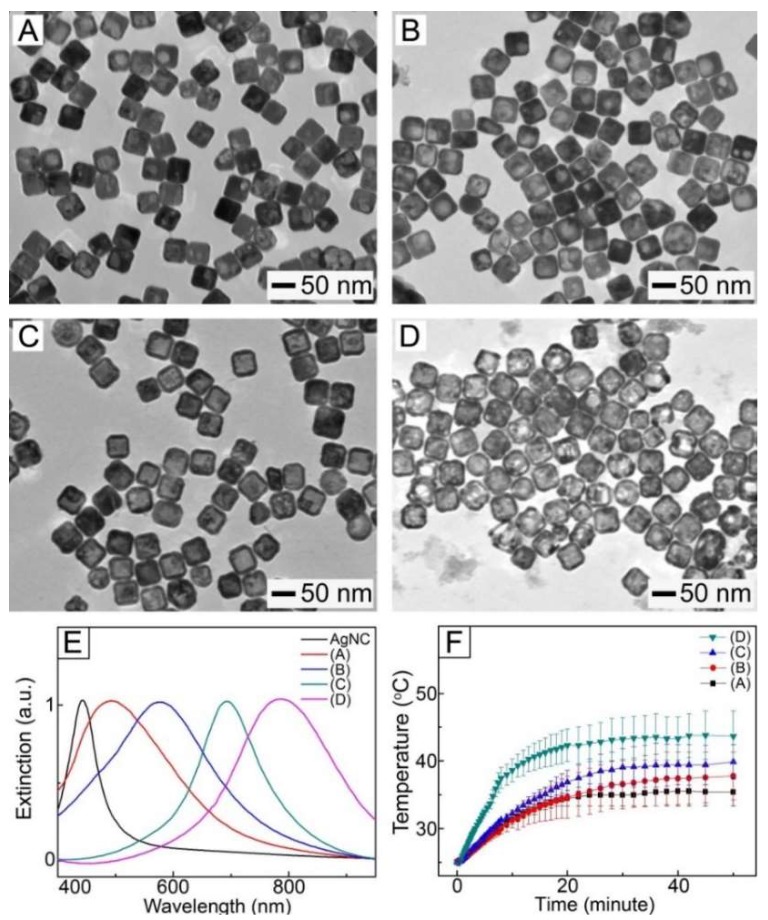
(**A**–**D**) TEM images of the particles obtained after the reaction of AgNCs with different amounts of HAuCl_4_ solution: (**A**) 0.06, (**B**) 0.08, (**C**) 0.15 and (**D**) 0.2 mL. (**E**) UV–VIS–NIR extinction spectra for the particles of (**A**–**D**) in DI water. (**E**) UV–VIS–NIR extinction spectra for the particles of (**A**–**D**) and AgNCs in DI water. (**F**) Change in temperature for the particles of (**A**–**D**) in DI water with a concentration of 0.1 mg/mL under NIR light irradiation (1 W/cm^2^, 808 nm).

AuNCs were synthesized using a galvanic replacement reaction between AgNCs (Figure S1, Supplementary Materials) and HAuCl_4_. When a very small amount of HAuCl_4_ solution was added to an aqueous solution of AgNCs, the replacement reaction was activated at a specific site on the surface of each AgNC, generating a hole (Figure 1A). As the reaction proceeded, the hole worked as an anode, oxidizing Ag atoms. Consequently, the released electrons moved to the surface of the AgNCs for reducing HAuCl_4_^−^, leading to an epitaxial growth of Au atoms on the AgNCs [28]. As the Au layer formed, the holes functioned as a site for the dissolution of Ag, transforming the AgNCs into a hollow structure (Figure 1B). When the amount of the HAuCl_4_ solution was increased, the hollow interior would be larger in size, forming Au-Ag nanoboxes with a uniform wall (Figure 1C). The Ag atoms in the nanoboxes would be selectively removed with the further addition of HAuCl_4_, eventually leading to the formation of AuNCs with a porous structure (Figure 1D). 

Figure 1E shows UV-VIS–NIR extinction spectra for the samples of Figure 1A–D dispersed in DI water, indicating that their surface plasmon resonance (SPR) band was dependent on the amount of HAuCl_4_ used. When a larger amount of HAuCl_4_ was used, the extinction peak appeared at a longer wavelength. For example, the sample of Figure 1B exhibited an extinction peak at 575 nm, while the SPR peak for the solution of AuNCs (Figure 1D) was red-shifted to 790 nm in the NIR region. This shift in SPR band had a direct impact on the photothermal ability of the samples. Figure 1F shows the change in temperature of each sample during exposure to 1 W/cm^2^ NIR light irradiation with a wavelength of 808 nm, indicating that the temperature of all the samples was increased by NIR light irradiation. However, the exact profiles differed. The sample of Figure 1A exhibited a temperature increase to 35 °C, while the increase in temperature up to 37 °C was seen in the sample of Figure 1B. The largest temperature rise was observed for the sample of Figure 1D because the SPR peak of this sample well matched the central wavelength of the laser source [29]. This result indicates that AuNCs could be a photothermal agent responding to NIR light. 

**Figure 2:**
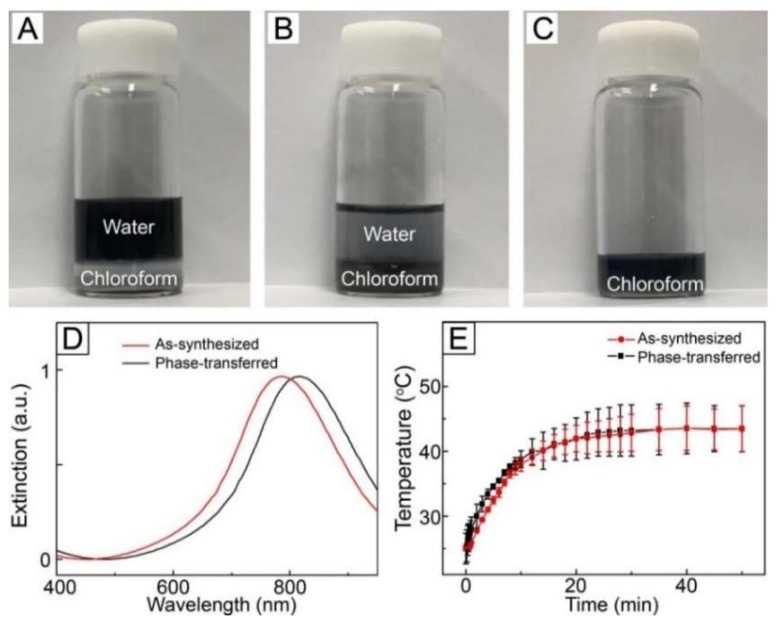
Photographs of AuNC solutions: (**A**) a mixture solution of AuNCs and PEG-thiol in a co-solvent of DI water and chloroform before vigorous agitation, (**B**) a mixture solution of AuNCs and PEG-thiol in a co-solvent of DI water and chloroform after vigorous agitation, and (**C**) a solution of phase-transferred AuNCs in chloroform. (**D**) UV–VIS–NIR extinction spectra for as-synthesized AuNCs in DI water and phase-transferred AuNCs in chloroform. (**E**) Time-dependent change in temperature for the as-synthesized AuNC solution in DI water and the phase-transferred AuNC solution in chloroform under NIR light irradiation (1 W/cm^2^, 808 nm).

For the fabrication of NIR light-sensitive fibrous system, loading of AuNCs into polymeric fibers is required. To accomplish this, the particles should be dispersed in organic media without aggregation. We phase-transferred the as-synthesized AuNCs from the aqueous phase to the organic using thiolated PEG as a transfer agent. PEG-thiol was first introduced to an aqueous solution containing the as-synthesized AuNCs, followed by chloroform. As shown in Figure 2A, the organic phase and the aqueous phase containing the particles were phase-separated because of their immiscibility. After vigorous agitation, most of the particles transferred from the water phase to the chloroform in 30 min (Figure 2B) [30]. This successful phase transfer was due to the surface modification caused by the strong affinity between the surface of AuNCs and the thiol group [30,31]. After extraction, centrifugation, and washing, the particles were re-dispersed in fresh chloroform to make a W/O emulsion for electrospinning (Figure 2C). 

Figure 2D shows UV–VIS–NIR extinction spectra for the as-synthesized AuNCs dispersed in DI water and the phase-transferred AuNCs dispersed in chloroform. No broadening of the SPR band was observed for the latter as compared with the former, which implies that aggregation among the AuNCs and change in their morphology did not happen during the phase transfer process. On the other hand, the phase transfer caused a red-shift in SPR band and the extinction peak moved to 815 nm in the NIR region, which was a result of change in the refractive index of the local environment surrounding the AuNCs [32]. 

We tested the photothermal conversion property of the phase-transferred AuNCs by exposing a solution of the phase-transferred particles in chloroform to NIR light irradiation. As shown in Figure 2E, the temperature of the sample increased in response to NIR light irradiation and its time-dependent temperature profile was similar to that of the aqueous solution of the as-synthesized AuNCs, which suggest that the photothermal ability of AuNCs was still kept after the phase transfer process. We also measured the optical property of the phase-transferred AuNCs after NIR light treatment. No change in the SPR band was observed after the treatment (Figure S2, Supplementary Materials), implying that the particles maintained their morphology without aggregation after NIR light irradiation. These results reveal that the phase-transferred AuNCs had excellent photostability. 

**Figure 3:**
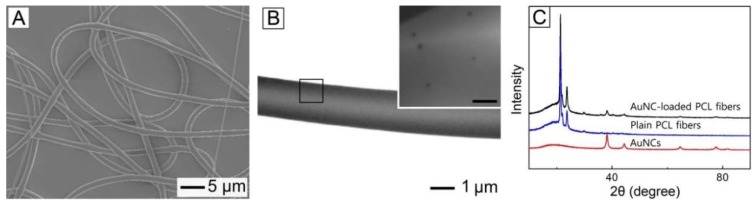
(**A**) SEM and (**B**) TEM images of PCL fibers loaded with AuNCs. The scale bar in the inset is 300 nm. (**C**) XRD patterns for AuNCs, plain PCL fibers, and AuNC-loaded PCL fibers.

The phase-transferred AuNCs were dispersed in a W/O emulsion prepared for fabrication of NIR light-sensitive polymeric fibers. Figure 3A shows a SEM image of the fibers produced by electrospinning the W/O emulsion. The used W/O emulsion contained the phase-transferred AuNCs of 0.1 wt% without any inclusion of drug. The produced fibers had a smooth surface and their average diameter was 2.84 ± 0.12 μm. Figure 3B shows a TEM image of a single fiber of Figure 3A. It was found that a hollow interior, which can be a reservoir of hydrophilic drug, was formed in the core of the fiber, which was a result of a big difference in the surface energy between water droplet phase and chloroform one that were present within the W/O emulsion [21]. A magnified image of the region, marked by a black box, is shown in the inset, demonstrating that the phase-transferred AuNCs were well distributed within the fiber. Figure 3C shows XRD patterns for the phase-transferred AuNCs, plain PCL fibers, and the fibers of Figure 3A. The XRD pattern from the sample of Figure 3A contained the peaks generated by the AuNCs as well as those corresponding to the plain PCL fibers. This result also confirms that the nanoparticles were successfully loaded into the fibers. 

**Figure 4:**
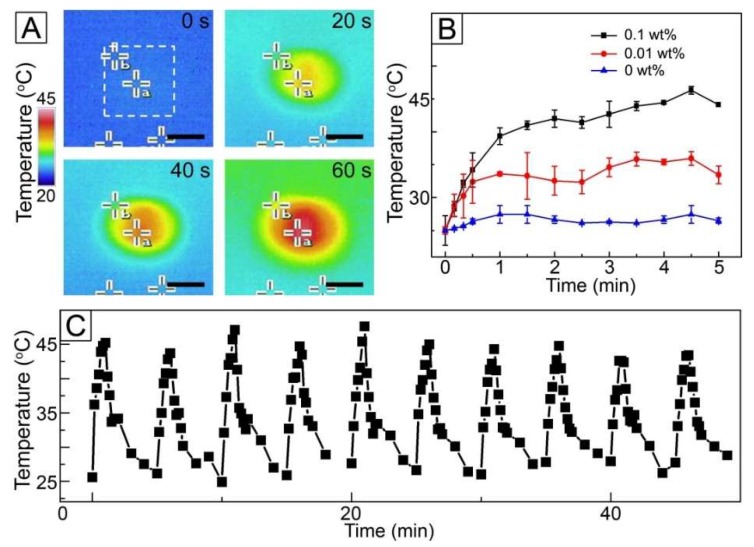
(**A**) Time-lapse thermal infrared images of AuNC-loaded fibers under NIR light irradiation (1 W/cm^2^, 808 nm). The scale bar is 0.5 cm. (**B**) Change in temperature for the fibers, which were produced from the W/O emulsions with different concentration of AuNCs, under NIR light (1 W/cm^2^, 808 nm). (**C**) Time-dependent temperature curve for AuNC-loaded fibers under on–off switching of NIR light (1 W/cm^2^, 808 nm).

To investigate a NIR light sensitivity of the fibers loaded with AuNCs, the fibres were exposed to NIR light irradiation and then their change in temperature observed. Figure 4A shows the time-lapse thermal infrared photographs of the fibers of Figure 3A under NIR light with a power density of 1 W/cm^2^. When the fibers were exposed to NIR irradiation, a drastic change in color from blue to red was observed at the center of the images (marked with a white box) where the fibers were placed. This change in color implies that the temperature of the sample increased due to the loading of AuNCs with photothermal conversion capability. Change in the concentration of AuNCs in the W/O emulsion had an influence on the heat generation property of the fibers. As shown in Figure 4B, the sample without the inclusion of AuNCs exhibited no significant change in temperature under NIR light irradiation, whereas the use of the W/O emulsion with 0.01 wt% AuNCs allowed the temperature rise to 33 °C. When the concentration increased to 0.1 wt%, the temperature reached 45 °C. This result was because the higher concentration of AuNCs would lead to loading of the more amount of the particles into the fibers, which resulted in stronger heat generation. 

The NIR light-triggered heat generation could reversibly happen when NIR light irradiation was applied in an on–off manner, as shown in Figure 4C. The treatment with NIR light for 1 min changed the temperature of the sample of Figure 3A from 26 to 45 °C, and the temperature returned to 26 °C after the light remained off for 4 min. This NIR light-sensitive manner in temperature change was still kept when the light was switched on and off ten times under the same condition, which indicates that the fibers would be useful for repeated hyperthermia treatment. 

We evaluated the anticancer ability of the fibers against SK-BR-3 cells. Before the evaluation, we checked the cytotoxicity of NIR light to the cells. Figure S3A (Supplementary Materials) shows the viability of SK-BR-3 cells after exposure to 1 W/cm^2^ NIR light, determined using a WST-1 assay. The viability of the cells was >90% for all the conditions compared with the control without the treatment of NIR light. In addition, we tested the cytotoxicity of the phase-transferred AuNCs and the result reveals that the particles were biocompatible (Figure S3B, Supplementary Materials). These results suggest that their effects on the anticancer ability of the fibers were negligible. 

**Figure 5:**
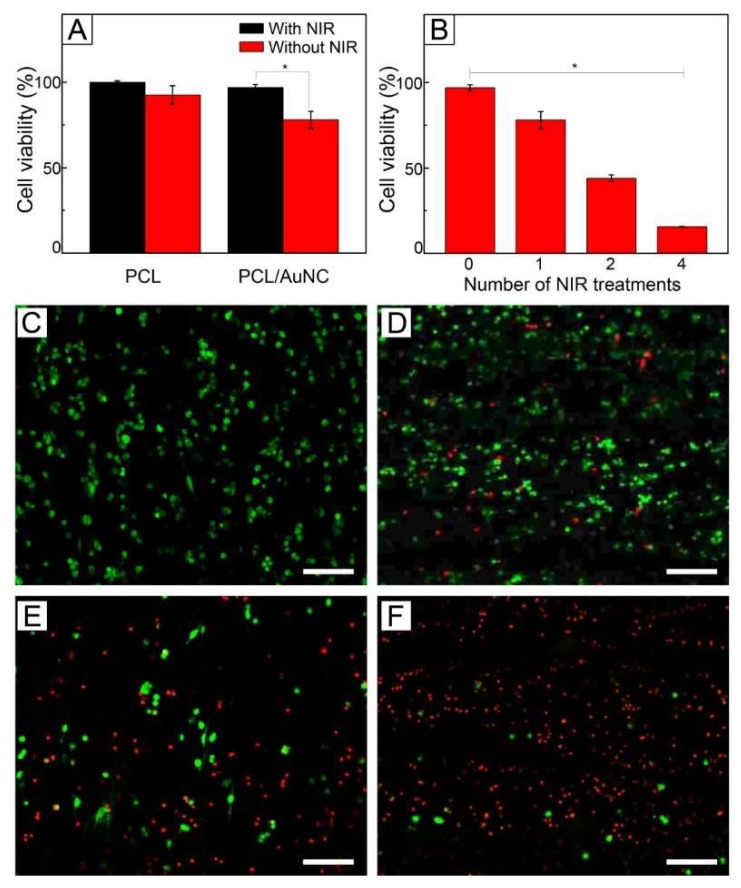
(**A**) Viability of SK-BR-3 cells treated with plain PCL and AuNC-loaded PCL fibers without and with NIR irradiation (1 W/cm^2^, 808 nm). (**B**) Viability of SK-BR-3 cells treated with AuNC-loaded PCL fibers under on–off switching of NIR light irradiation (1 W/cm^2^, 808 nm). * indicates *p* < 0.01. (**C**–**F**) CLS micrographs showing live (green) and dead (red) SK-BR-3 cells treated with AuNC-loaded PCL fibers under different numbers of cycles of 1 W/cm^2^ NIR light irradiation: (**C**) Zero, (**D**) one, (**E**) two, and (**F**) four cycles. The scale bars are 100 μm.

Figure 5A shows the viabilities of SK-BR-3 cells treated with plain PCL fibers and AuNC-loaded PCL fibers of Figure 3A. Under no exposure to NIR light, both the samples exhibited cell viabilities >90%. A similar result was also observed with FB cells (Figure S4, Supplementary Materials). The high viabilities indicate that the fibers are biocompatible. On the other hand, when irradiated with NIR light, only the fibers loaded with AuNCs had a lower cell viability (78%). This decrease in cell viability was because the photothermal conversion generated by the loaded particles increased the temperature of the sample to 45 °C for hyperthermia, and consequently damaged the cells. However, many cells were still alive, even though they might be weakened. 

This hyperthermia effect could be strengthened by increasing the number of cycles of NIR light irradiation. Figure 5B shows the cell viability with respect to the number of NIR light treatments, demonstrating that the value of viability decreased as the number of the treatments increased. Two cycles of NIR light treatment yielded a cell viability of 44%, while four cycles of the treatment reduced the value to 16%. This decrease in viability can be explained by the shock effect [33]. The repeated, rapid temperature change due to multiple cycles of on–off operation of NIR light irradiation could damage more the already weakened cells, resulting in more destruction of the cells. Live/dead assay also showed the decrease in cell viability. Figure 5C shows a CLS micrograph of SK-BR-3 cells treated with AuNC-loaded fibers under no NIR light, which exhibited only a bright green fluorescence. This result suggests that all the cancer cells were still alive. On the other hand, the red fluorescence became more visible as the number of cycles of NIR light treatment increased (Figure 5D–F), implying cell death was induced by the applications of NIR light-driven hyperthermia.

**Figure 6:**
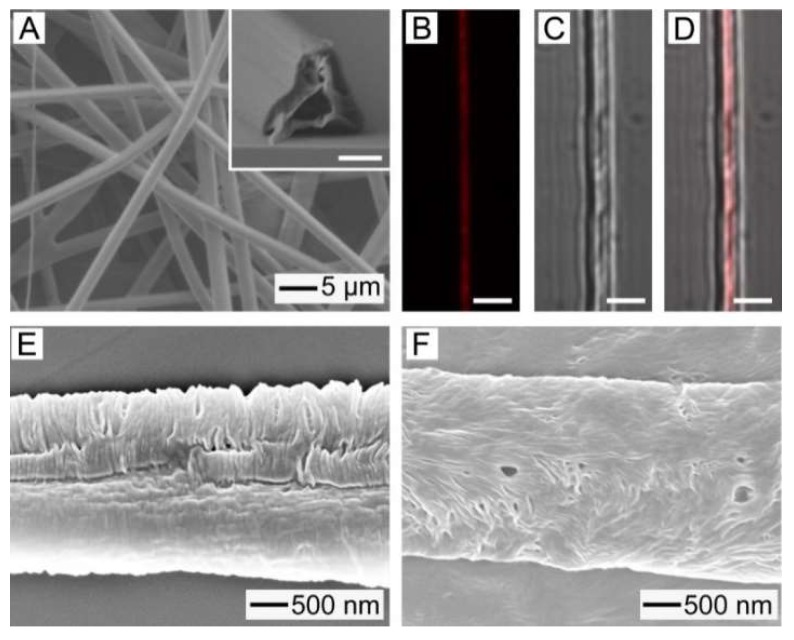
(**A**) SEM image of AuNC-loaded fibers containing the fatty acid mixture and DOX, which were produced using a W/O emulsion of *f_fatty_* = 0.05. The inset scale is 1 μm. (**B**–**D**) CLS micrographs showing the fluorescence from DOX loaded in the as-spun fiber. The scales are 3 μm. (**E**,**F**) SEM images of AuNC-loaded fibers after exposure to 1 W/cm^2^ NIR light. The fibers were made using W/O emulsions of: (**E**) *f_fatty_* = 0.05 and (**F**) *f_fatty_* = 0.1.

However, the use of only NIR light-induced hyperthermia cannot achieve a satisfactory therapeutic efficacy because of the non-uniform distribution of heat generated within a tumor [13]. Furthermore, residual cancer cell survival after the treatment can cause metastasis or recurrence [20]. To improve the anticancer activity of the AuNC-loaded fibers, we additionally loaded a chemotherapeutic drug, DOX, into the fibers together with a biocompatible, phase-changeable fatty acid that could induce a NIR-light triggered release of the drug [21,22,26]. Figure 6A shows a SEM image of AuNC-loaded PCL fibers with the inclusion of the drug and the fatty acid mixture of LA and SA, which were produced by electrospinning a W/O emulsion containing DOX in the water phase and the fatty acid mixture (*f_fatty_* = 0.05) in the oil phase. The average diameter of the fibers was 2.88 ± 0.14 μm, which was similar to that for the sample of Figure 3A. A cross-sectional SEM image is shown in the inset, demonstrating that the fibers had a hollow interior and a smooth surface. These results indicate that the addition of the fatty acid mixture and the drug did not affect the formation of fibers or their structure. Figure 6B–D show CLS images of the single fiber of Figure 6A. Red fluorescence generated from DOX is observed in the fluorescence image of Figure 6B, suggesting that the drug molecules were well loaded in the fibers. The merged image (Figure 6D) with its corresponding optical image (Figure 6C) clearly demonstrates that DOX was placed in the central region of the fiber. Taken together with the inset of Figure 6A, this result indicates that the drug molecules were encapsulated in the hollow core of the fibers. 

To check the inclusion of the fatty acid mixture into the fibers, DSC measurement was conducted. The fibers of Figure 6A exhibited two sharp peaks at 39 and 59 °C (Figure S5, Supplementary Materials), which corresponded to the melting points of the fatty acid mixture and PCL, respectively [22]. This result reveals the successful loading of the fatty acid mixture into the fibers. After introduction of 1 W/cm^2^ NIR light irradiation to increase the temperature of the fibers to 45 °C, the peak at 39 °C given by the fatty acid mixture disappeared (Figure S5), indicating that the mixture was removed from the fibers. This removal was a result of NIR light-induced photothermal effect. Upon exposure to NIR light, a photothermal conversion was caused by the loaded AuNCs, increasing the local temperature of the sample above the melting point of the fatty acid mixture and melting out the mixture from the fibers. Consequently, small pores were generated in the sheath of the fibers, as shown in Figure 6E. The size and density of the formed pores increased with *f_fatty_* (Figure 6F) because of the inclusion of the larger amount of the mixture in the fibers. 

**Figure 7:**
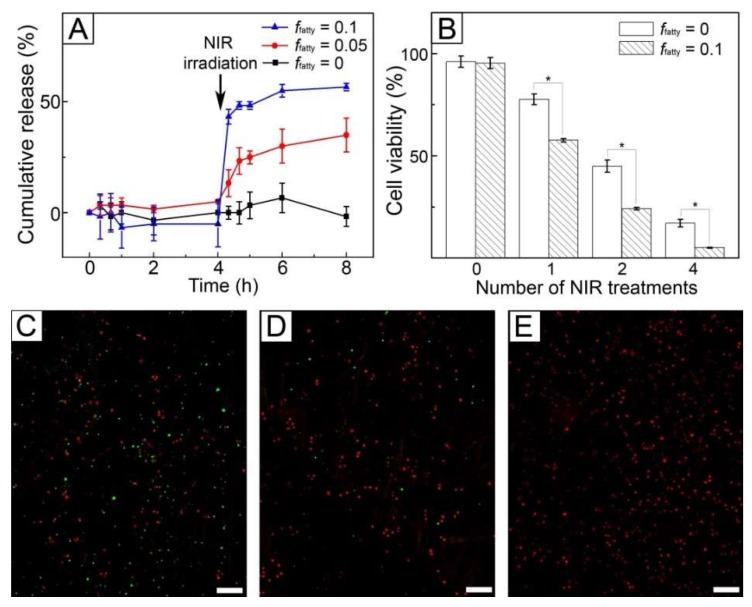
(**A**) Release profiles of DOX from AuNC-loaded fibers containing the fatty acid mixture and the drug. The cumulative release (%) percentage was defined as a ratio of the accumulated amount of released drug at a given time to the initial amount loaded. (**B**) Viability of SK-BR-3 cells treated with AuNC-loaded fibers containing the fatty acid mixture and DOX under different numbers of cycles of 1 W/cm^2^ NIR light irradiation. The fibers were made using W/O emulsions of *f_fatty_* = 0 and 0.1. * indicates *p* < 0.01. (**C**–**E**) CLS micrographs showing live (green) and dead (red) SK-BR-3 cells treated with the fibers of *f_fatty_* = 0.1 under different numbers of cycles of 1 W/cm^2^ NIR light irradiation: (**C**) one, (**D**) two, and (**E**) four cycles. The scale bars are 100 μm.

Figure 7A shows the release profiles of DOX from the fibers produced from the W/O emulsions with different values of *f_fatty_*. The sample with no inclusion of the fatty acid mixture (*f_fatty_* = 0) did not release the drug molecules over 4 h without NIR light treatment. A similar result was also observed for the fibers produced with the use of the W/O emulsion of *f_fatty_* = 0.05 because the fatty acid mixture present in the solid state blocked the diffusion-out of the encapsulated drug molecules. When 1 W/cm^2^ NIR light was irradiated for 5 min, the sample of *f_fatty_* = 0 still did not release the drug molecules, whereas the fibers of *f_fatty_* = 0.05 allowed the release of 33% DOX for 4 h since NIR light was irradiated. The release in the latter case resulted from the formation of the pores in response to NIR light, as visualized in Figure 6E. The formation of the pores caused by the melting out of the loaded fatty acid could increase the surface area of the fibers, allowing an easy uptake of the PBS buffer into the fibers and a generation of effective paths for the preloaded drug molecules to be released quickly [22]. This NIR light-triggered release could be controlled by varying the value of *f_fatty_*. When *f_fatty_* increased to 0.1, the release of 55% DOX was achieved over the same period. This difference was attributed to the larger size and density of the generated pores for the sample of *f_fatty_* = 0.1 (Figure 7F), which increased surface area [21] and consequently led to the faster release of the drug.

Figure 7B shows the viability of SK-BR-3 cells treated with the fibers of *f_fatty_* = 0 and 0.1 in Figure 7A. When the fibers were not exposed to NIR light, the cell viabilities were above 90%. In the case of FB cells, the similar result was also observed (Figure S4). These results were because the fibers were biocompatible and the drug encapsulated in their core was not released, as indicated by the result of *in vitro* release in Figure 7A. When NIR light was irradiated, the cell viability was reduced for both the systems. However, their anticancer ability was different. The cell viability for the system with *f_fatty_* = 0 was similar to that of Figure 5B because the loaded drug molecules were not released (Figure 7A) and consequently only hyperthermia occurred. In the case of the system with *f_fatty_* = 0.1, the cell viability decreased to 57% after one cycle of NIR light, which was lower than the value (77%) of the fibers with *f_fatty_* = 0 with the use of only hyperthermia. This decrease in cell viability was due to the synergistic effect of the drug release and hyperthermia. The effect was much stronger with the increase of the number of cycles of NIR light treatment. Two cycles of NIR irradiation reduced the cell viability to 24%, and four cycles led to a cell viability of 4%. This cell death was also confirmed using a live/dead assay (Figure 7C–E), demonstrating that the majority of the cells were dead after four cycles of the treatment, as shown in Figure 7E.

## 4. Conclusions

A fibrous system for synergistic cancer therapy was successfully developed using a simple emulsion electrospinning technique. The system consisted of AuNC-incorporated PCL fibers encapsulating an anticancer drug, DOX, in their core and loading a phase-changeable fatty acid in their sheath. The excellent photothermal ability and photostability of AuNCs led to the repeated, significant heating of the fibers in response to on–off switching of NIR light irradiation. When the irradiation increased the temperature of the system to a window suitable for hyperthermia, the death of cancer cells was observed. The NIR light-induced heat generation also simultaneously allowed the release of the drug molecules through the pores in the fibers generated by the melting of the loaded fatty acid. The combination of this NIR light-triggered drug release with the repeated hyperthermia treatment exhibited excellent therapeutic efficacy in an in vitro model. Thus, the fibers have potential in synergistic cancer therapy with a combination of NIR-triggered hyperthermia and chemotherapy. 

## Supplementary Materials

The following are available online at http://www.mdpi.com/1999-4923/11/2/60/s1, Figure S1. TEM image of AgNCs used for synthesis of AuNCs. Figure S2. UV-vis-NIR extinction spectra for phase-transferred AuNCs in chloroform before and after exposure to NIR light for 50 min. Figure S3. Viability of SK-BR-3 cells treated with: A) NIR light and B) phase-transferred AuNCs. Figure S4. Viability of FB cells treated with plain PCL fibers, AuNC-incorporated PCL fibers, AuNC-incorporated PCL fibers loaded with DOX, and AuNC-incorporated PCL fibers with loading of the fatty acid mixture and DOX. Figure S5. DSC thermograms for AuNC-loaded fibers produced with a W/O emulsion of *f_fatty_* =0.05 before and after NIR irradiation.
