# Supplementary material for: Gold Nanocage-Incorporated Poly(ε-Caprolactone) (PCL) Fibers for Chemophotothermal Synergistic Cancer Therapy
Source: Pharmaceutics. 2019 Feb 1;11(2):60. doi: 10.3390/pharmaceutics11020060 (PMC6410212; doi:10.3390/pharmaceutics11020060)
Supplement: Supplementary file 1 [file pharmaceutics-11-00060-s001.pdf]

pharmaceutics

MDPI

Supplementary Materials: Gold Nanocage-Incorporated Poly(ε-Caprolactone) (PCL) Fibers for Chemophotothermal Synergistic Cancer Therapy

Ju Hyang Park, Hojun Seo, Da In Kim, Ji Hyun Choi, Jin Ho Son, Jongbok Kim, Geon Dae Moon and Dong Choon Hyun

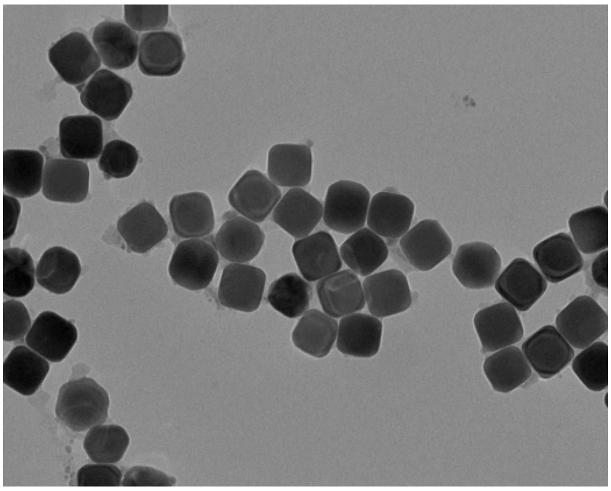

Figure S1. TEM image of AgNCs used for synthesis of AuNCs.

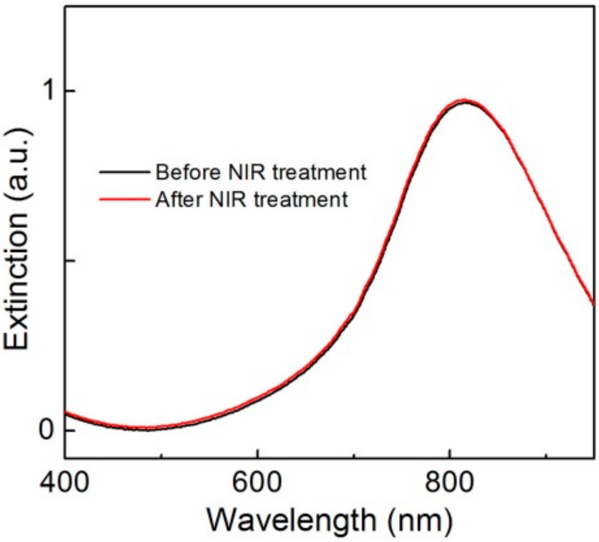

Figure S2. UV-VIS-NIR extinction spectra for phase-transferred AuNCs in chloroform before and after exposure to NIR light (1 W/cm^2, 808 nm) for 50 min.

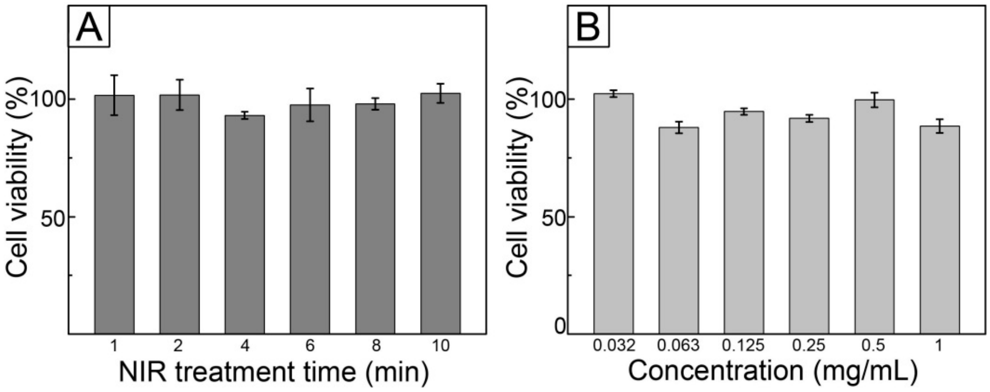

Figure 3. Viability of SK-BR-3 cells treated with: A) NIR light (1 W/cm^2, 808 nm) and B) phase-transferred AuNCs.

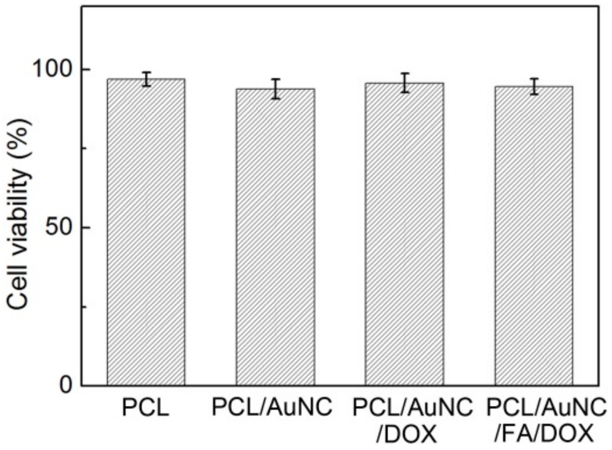

Figure S4. Viability of FB cells treated with plain PCL fibers (PCL), AuNC-incorporated PCL fibers (PCL/AuNC), AuNC-incorporated PCL fibers loaded with DOX (PCL/AuNC/DOX), and AuNC-incorporated PCL fibers with loading of the fatty acid mixture and DOX (PCL/AuNC/FA/DOX).

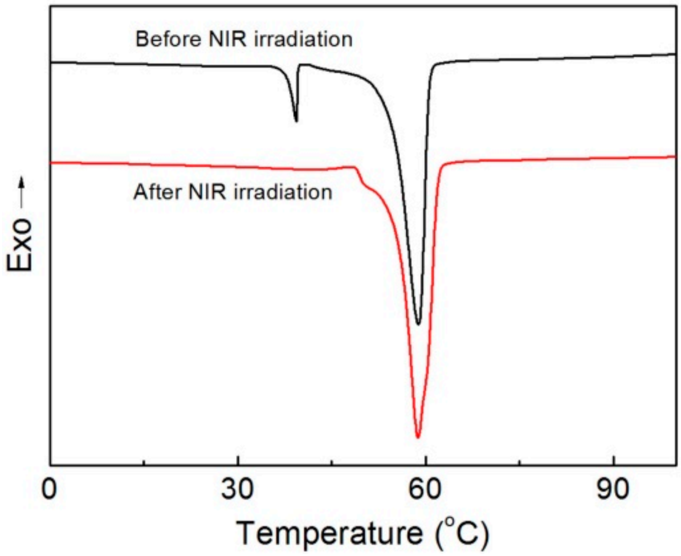

Figure S5. DSC thermograms for AuNC-loaded fibers produced with a W/O emulsions of f_{fatty}=0.05 before and after NIR irradiation (1 W/cm^2, 808 nm).
